# 1-Benzyl-2-(1*H*-indol-3-yl)-5-oxo­pyrrolidine-2-carbonitrile

**DOI:** 10.1107/S1600536807067670

**Published:** 2008-01-04

**Authors:** Rafael Tamazyan, Ayvazyan Armen, Martirosyan Ashot, Gasparyan Sahak, Raymond Schinazi

**Affiliations:** a Molecule Structure Research Center, National Academy of Sciences RA, Azatutyan ave. 26, 375014 Yerevan, Republic of Armenia; b Institute of Fine Organic Chemistry, National Academy of Sciences RA, Azatutyan ave. 26, 375014 Yerevan, Republic of Armenia; c Emory University School of Medicine, Veterans Affairs Medical Center, 1670 Clairmont Road, 151-H Decatur, Georgia 30033-4004, USA

## Abstract

In the title compound, C_20_H_17_N_3_O, a potential anti-human immunodeficiency virus type 1 (HIV-1) non-nucleoside reverse-transcriptase inhibitor, the pyrrolidine ring has an envelope conformation. In the crystal structure, adjacent mol­ecules are connected into infinite chains *via* an N—H⋯O hydrogen bond.

## Related literature

For details of the synthesis, see: Martirosyan *et al.* (2000 ▶, 2004 ▶). For details of the pharmacological properties of compounds of this family, see: De Clercq (1996 ▶). For the crystal structures of some analogs of the title compound, see: Karapetyan *et al.* (2002 ▶); Tamazyan *et al.* (2002 ▶).
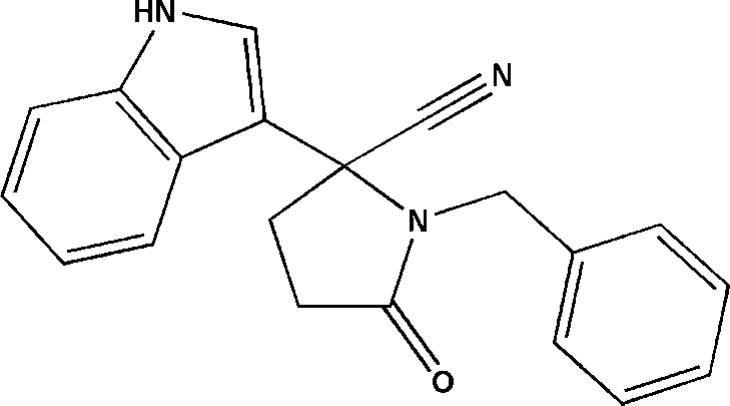


## Experimental

### 

#### Crystal data

C_20_H_17_N_3_O
*M* *_r_* = 315.37
Triclinic, 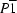

*a* = 7.5781 (15) Å
*b* = 9.4521 (19) Å
*c* = 12.409 (3) Å
α = 78.02 (3)°
β = 83.05 (3)°
γ = 69.68 (3)°
*V* = 814.2 (3) Å^3^
*Z* = 2
Mo *K*α radiation
μ = 0.08 mm^−1^
*T* = 293 (2) K
0.4 × 0.3 × 0.2 mm

#### Data collection

Enraf–Nonius CAD-4 diffractometer
Absorption correction: none
9470 measured reflections
4735 independent reflections
3450 reflections with *I* > 2σ(*I*)
*R*_int_ = 0.020
3 standard reflections frequency: 180 min intensity decay: none

#### Refinement

*R*[*F* ^2^ > 2σ(*F* ^2^)] = 0.047
*wR*(*F* ^2^) = 0.130
*S* = 1.02
4735 reflections
285 parameters
H atoms treated by a mixture of independent and constrained refinement
Δρ_max_ = 0.18 e Å^−3^
Δρ_min_ = −0.20 e Å^−3^

### 

Data collection: *CAD-4 Manual* (Enraf–Nonius, 1988 ▶); cell refinement: *CAD-4 Manual*; data reduction: *HELENA* (Spek, 1997 ▶); program(s) used to solve structure: *SHELXS97* (Sheldrick, 1997 ▶); program(s) used to refine structure: *SHELXL97* (Sheldrick, 1997 ▶); molecular graphics: *SHELXTL* (Bruker 2000 ▶) and *ORTEPII* (Johnson, 1976 ▶); software used to prepare material for publication: *SHELXTL*.

## Supplementary Material

Crystal structure: contains datablocks global, I. DOI: 10.1107/S1600536807067670/su2039sup1.cif

Structure factors: contains datablocks I. DOI: 10.1107/S1600536807067670/su2039Isup2.hkl

Additional supplementary materials:  crystallographic information; 3D view; checkCIF report

## Figures and Tables

**Table 1:** Hydrogen-bond geometry (Å, °)

*D*—H⋯*A*	*D*—H	H⋯*A*	*D*⋯*A*	*D*—H⋯*A*
N11—H11⋯O6^i^	0.90 (2)	2.01 (2)	2.866 (2)	158 (2)

Symmetry code: (i) 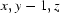.

**Fig. 1.**
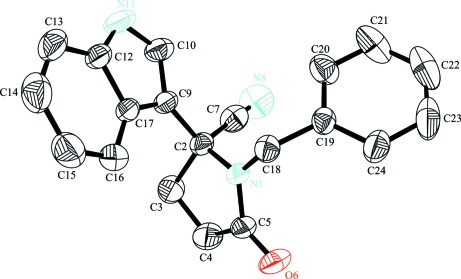
A view of molecular structure of the title compound, showing the atomic numbering scheme and displacement ellipsoids drawn at the 50% probability level. H atoms are omitted for clarity.

**Fig. 2.**
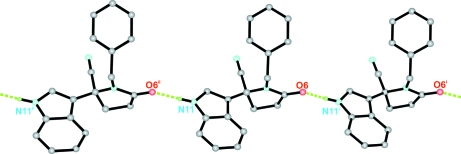
partial view of the crystal packing showing the formation of the infinite chain of molecules formed via hydrogen bonding [for clarifity only H atoms participating in bonding are depicted]. Symmetry codes: (i) x, 1 + y, z; (ii) x, -1 + y, z.

## supplementary crystallographic information

### Comment

Our interest in the X-ray structural investigation of the title compound was stimulated by its potential HIV-1 RT inhibition properties. Compounds of this type belong to family of non-nucleoside reverse transcriptase inhibitors (NNRTIs).

A view of the molecular structure of the title compound is given in Fig. 1. A l l the bond distances in the molecule are in good agreement with their mean statistical values, except bond C2—C7 which is relatively short, 1.493 (2) Å. We believe that this shortening is caused by the inductive effect of the carbonitryl group C7 ≡N11.

In the crystal structure infinite chains along [010] direction are formed *via* an intermolecular N—H···O hydrogen bond (see Fig. 2 and Table 1).

### Experimental

The title compound was synthesized by the cycloalkylation of N1-benzyl-N1-cyano(1*H*-3-indolyl)methyl-3-chloropropanamide in the phase-transfer catalysis condition (Martirosyan *et al.*, 2000, 2004). The compound as synthesized is a racemic mixture. Colorless crystals, suitable for X-ray analysis, were grown from a methanol solution of the compound.

### Refinement

The H-atoms were located from difference Fourier syntheses and freely refined: N—H = 0.90 (2) Å; C—H = 0.94 (2) - 1.02 (2) Å.

### Crystal data

**Table:**

C_20_H_17_N_3_O	*Z* = 2
*M**_r_* = 315.37	*F*_000_ = 332
Triclinic, *P*1	*D*_x_ = 1.286 Mg m^−^^3^
Hall symbol: -P 1	Mo *K*α radiation λ = 0.71073 Å
*a* = 7.5781 (15) Å	Cell parameters from 22 reflections
*b* = 9.4521 (19) Å	θ = 14.2–17.5º
*c* = 12.409 (3) Å	µ = 0.08 mm^−^^1^
α = 78.02 (3)º	*T* = 293 (2) K
β = 83.05 (3)º	Prism, colourless
γ = 69.68 (3)º	0.4 × 0.3 × 0.2 mm
*V* = 814.2 (3) Å^3^	

### Data collection

**Table:**

Enraf–Nonius CAD-4 diffractometer	*R*_int_ = 0.020
Radiation source: fine-focus sealed tube	θ_max_ = 30.0º
Monochromator: graphite	θ_min_ = 1.7º
*T* = 293(2) K	*h* = −10→10
θ/2θ scans	*k* = −13→13
Absorption correction: none	*l* = −17→17
9470 measured reflections	3 standard reflections
4735 independent reflections	every 180 min
3450 reflections with *I* > 2σ(*I*)	intensity decay: none

### Refinement

**Table:**

Refinement on *F*^2^	Secondary atom site location: difference Fourier map
Least-squares matrix: full	Hydrogen site location: inferred from neighbouring sites
*R*[*F*^2^ > 2σ(*F*^2^)] = 0.047	H atoms treated by a mixture of independent and constrained refinement
*wR*(*F*^2^) = 0.130	*w* = 1/[σ^2^(*F*_o_^2^) + (0.0649*P*)^2^ + 0.0907*P*] where *P* = (*F*_o_^2^ + 2*F*_c_^2^)/3
*S* = 1.02	(Δ/σ)_max_ < 0.001
4735 reflections	Δρ_max_ = 0.18 e Å^−^^3^
285 parameters	Δρ_min_ = −0.20 e Å^−^^3^
Primary atom site location: structure-invariant direct methods	Extinction correction: none

### Special details

**Table:**

Geometry. All e.s.d.'s (except the e.s.d. in the dihedral angle between two l.s. planes) are estimated using the full covariance matrix. The cell e.s.d.'s are taken into account individually in the estimation of e.s.d.'s in distances, angles and torsion angles; correlations between e.s.d.'s in cell parameters are only used when they are defined by crystal symmetry. An approximate (isotropic) treatment of cell e.s.d.'s is used for estimating e.s.d.'s involving l.s. planes.
Refinement. Refinement of *F*^2^ against ALL reflections. The weighted *R*-factor *wR* and goodness of fit *S* are based on *F*^2^, conventional *R*-factors *R* are based on *F*, with *F* set to zero for negative *F*^2^. The threshold expression of *F*^2^ > σ(*F*^2^) is used only for calculating *R*-factors(gt) *etc*. and is not relevant to the choice of reflections for refinement. *R*-factors based on *F*^2^ are statistically about twice as large as those based on *F*, and *R*- factors based on ALL data will be even larger.

### Fractional atomic coordinates and isotropic or equivalent isotropic displacement parameters (Å^2^)

**Table:**

	*x*	*y*	*z*	*U*_iso_*/*U*_eq_	
N1	0.22574 (14)	0.71961 (10)	0.23198 (8)	0.0367 (2)	
C2	0.20774 (17)	0.64700 (12)	0.14116 (9)	0.0385 (2)	
C3	0.3119 (3)	0.72278 (16)	0.04282 (12)	0.0552 (4)	
H3A	0.448 (3)	0.655 (2)	0.0402 (14)	0.066 (5)*	
H3B	0.258 (2)	0.728 (2)	−0.0253 (16)	0.069 (5)*	
C4	0.2913 (3)	0.87637 (17)	0.07212 (14)	0.0642 (4)	
H4A	0.182 (3)	0.955 (3)	0.0430 (19)	0.096 (7)*	
H4B	0.408 (3)	0.908 (3)	0.049 (2)	0.102 (7)*	
C5	0.26566 (17)	0.85107 (13)	0.19576 (12)	0.0453 (3)	
O6	0.27772 (15)	0.93414 (11)	0.25649 (10)	0.0628 (3)	
C7	0.0051 (2)	0.69595 (15)	0.11645 (11)	0.0503 (3)	
N8	−0.1486 (2)	0.73626 (18)	0.09409 (14)	0.0761 (4)	
C9	0.28897 (17)	0.47561 (12)	0.16540 (9)	0.0379 (2)	
C10	0.2034 (2)	0.37431 (15)	0.15315 (11)	0.0480 (3)	
H10	0.076 (2)	0.3968 (19)	0.1273 (14)	0.063 (5)*	
H11	0.298 (3)	0.140 (2)	0.1869 (15)	0.073 (5)*	
N11	0.32401 (19)	0.22756 (13)	0.18058 (10)	0.0543 (3)	
C12	0.48874 (19)	0.23143 (13)	0.21285 (10)	0.0435 (3)	
C13	0.6498 (2)	0.11135 (15)	0.24876 (12)	0.0574 (4)	
H13	0.655 (2)	0.009 (2)	0.2546 (15)	0.068 (5)*	
C14	0.7955 (2)	0.14884 (19)	0.27645 (14)	0.0656 (4)	
H14	0.911 (3)	0.066 (2)	0.3034 (16)	0.079 (5)*	
C15	0.7826 (2)	0.30164 (19)	0.26931 (15)	0.0641 (4)	
H15	0.887 (3)	0.324 (2)	0.2894 (16)	0.078 (5)*	
C16	0.62465 (19)	0.42094 (15)	0.23300 (12)	0.0498 (3)	
H16	0.620 (2)	0.5269 (19)	0.2264 (14)	0.059 (4)*	
C17	0.47300 (17)	0.38670 (12)	0.20376 (9)	0.0379 (2)	
C18	0.20486 (17)	0.65739 (14)	0.34923 (10)	0.0395 (2)	
H18A	0.258 (2)	0.5459 (17)	0.3571 (12)	0.047 (4)*	
H18B	0.280 (2)	0.6986 (17)	0.3851 (13)	0.054 (4)*	
C19	0.00691 (16)	0.70242 (13)	0.40041 (9)	0.0376 (2)	
C20	−0.1177 (2)	0.63319 (19)	0.38244 (12)	0.0545 (3)	
H20	−0.077 (2)	0.557 (2)	0.3324 (15)	0.070 (5)*	
C21	−0.2973 (2)	0.6704 (3)	0.43232 (14)	0.0744 (5)	
H21	−0.385 (3)	0.624 (2)	0.4196 (19)	0.098 (7)*	
C22	−0.3538 (3)	0.7749 (2)	0.50150 (15)	0.0782 (6)	
H22	−0.484 (3)	0.800 (2)	0.5378 (19)	0.099 (7)*	
C23	−0.2317 (3)	0.8426 (2)	0.52187 (14)	0.0750 (5)	
H23	−0.264 (3)	0.914 (3)	0.5704 (19)	0.095 (7)*	
C24	−0.0502 (2)	0.80719 (16)	0.47138 (11)	0.0542 (3)	
H24	0.038 (2)	0.858 (2)	0.4834 (14)	0.065 (5)*	

### Atomic displacement parameters (Å^2^)

**Table:**

	*U*^11^	*U*^22^	*U*^33^	*U*^12^	*U*^13^	*U*^23^
N1	0.0439 (5)	0.0279 (4)	0.0399 (5)	−0.0152 (4)	0.0056 (4)	−0.0083 (3)
C2	0.0494 (6)	0.0315 (5)	0.0350 (5)	−0.0157 (5)	0.0037 (5)	−0.0062 (4)
C3	0.0742 (10)	0.0429 (7)	0.0425 (7)	−0.0210 (7)	0.0150 (7)	−0.0026 (5)
C4	0.0842 (11)	0.0384 (7)	0.0649 (9)	−0.0266 (7)	0.0159 (8)	0.0017 (6)
C5	0.0426 (6)	0.0272 (5)	0.0655 (8)	−0.0132 (4)	0.0051 (5)	−0.0089 (5)
O6	0.0691 (7)	0.0387 (5)	0.0905 (8)	−0.0253 (5)	0.0011 (6)	−0.0228 (5)
C7	0.0609 (8)	0.0433 (6)	0.0447 (7)	−0.0147 (6)	−0.0096 (6)	−0.0040 (5)
N8	0.0693 (9)	0.0762 (10)	0.0782 (10)	−0.0154 (7)	−0.0267 (8)	−0.0059 (8)
C9	0.0500 (6)	0.0304 (5)	0.0358 (5)	−0.0170 (5)	0.0045 (5)	−0.0089 (4)
C10	0.0624 (8)	0.0415 (6)	0.0490 (7)	−0.0263 (6)	−0.0024 (6)	−0.0121 (5)
N11	0.0788 (8)	0.0341 (5)	0.0594 (7)	−0.0295 (5)	0.0002 (6)	−0.0122 (5)
C12	0.0605 (7)	0.0309 (5)	0.0390 (6)	−0.0180 (5)	0.0111 (5)	−0.0089 (4)
C13	0.0738 (10)	0.0326 (6)	0.0532 (8)	−0.0100 (6)	0.0146 (7)	−0.0041 (5)
C14	0.0545 (9)	0.0536 (8)	0.0666 (10)	−0.0005 (7)	0.0081 (7)	0.0005 (7)
C15	0.0467 (8)	0.0635 (9)	0.0771 (11)	−0.0168 (7)	0.0016 (7)	−0.0070 (8)
C16	0.0471 (7)	0.0418 (6)	0.0622 (8)	−0.0188 (5)	0.0056 (6)	−0.0105 (6)
C17	0.0475 (6)	0.0299 (5)	0.0369 (5)	−0.0156 (4)	0.0097 (5)	−0.0095 (4)
C18	0.0411 (6)	0.0387 (6)	0.0377 (6)	−0.0114 (5)	−0.0004 (5)	−0.0090 (4)
C19	0.0429 (6)	0.0351 (5)	0.0307 (5)	−0.0093 (4)	0.0006 (4)	−0.0049 (4)
C20	0.0553 (8)	0.0719 (9)	0.0436 (7)	−0.0304 (7)	0.0050 (6)	−0.0144 (6)
C21	0.0521 (9)	0.1182 (16)	0.0534 (9)	−0.0380 (10)	0.0035 (7)	−0.0031 (9)
C22	0.0529 (9)	0.0940 (13)	0.0560 (9)	−0.0023 (9)	0.0154 (7)	0.0060 (9)
C23	0.0899 (13)	0.0588 (9)	0.0509 (8)	0.0010 (9)	0.0230 (8)	−0.0149 (7)
C24	0.0726 (9)	0.0435 (7)	0.0429 (7)	−0.0147 (6)	0.0073 (6)	−0.0138 (5)

### Geometric parameters (Å, °)

**Table:**

N1—C5	1.3542 (15)	C13—C14	1.370 (3)
N1—C18	1.4634 (16)	C13—H13	0.944 (18)
N1—C2	1.4775 (15)	C14—C15	1.398 (2)
C2—C7	1.493 (2)	C14—H14	0.99 (2)
C2—C9	1.4979 (16)	C15—C16	1.375 (2)
C2—C3	1.5562 (18)	C15—H15	0.96 (2)
C3—C4	1.521 (2)	C16—C17	1.4018 (18)
C3—H3A	1.009 (18)	C16—H16	0.976 (16)
C3—H3B	0.969 (18)	C18—C19	1.5076 (17)
C4—C5	1.502 (2)	C18—H18A	0.977 (15)
C4—H4A	0.95 (2)	C18—H18B	0.983 (16)
C4—H4B	1.02 (2)	C19—C20	1.3824 (19)
C5—O6	1.2269 (16)	C19—C24	1.3842 (17)
C7—N8	1.142 (2)	C20—C21	1.383 (2)
C9—C10	1.3695 (17)	C20—H20	0.989 (18)
C9—C17	1.4348 (18)	C21—C22	1.366 (3)
C10—N11	1.3689 (19)	C21—H21	0.95 (2)
C10—H10	0.994 (17)	C22—C23	1.365 (3)
N11—C12	1.3707 (19)	C22—H22	1.00 (2)
N11—H11	0.90 (2)	C23—C24	1.399 (2)
C12—C13	1.391 (2)	C23—H23	0.94 (2)
C12—C17	1.4120 (15)	C24—H24	0.986 (17)
			
C5—N1—C18	122.70 (10)	C14—C13—H13	122.7 (10)
C5—N1—C2	112.91 (10)	C12—C13—H13	120.0 (10)
C18—N1—C2	124.39 (9)	C13—C14—C15	121.35 (15)
N1—C2—C7	109.37 (10)	C13—C14—H14	119.6 (11)
N1—C2—C9	113.04 (10)	C15—C14—H14	119.1 (11)
C7—C2—C9	110.17 (11)	C16—C15—C14	121.65 (16)
N1—C2—C3	101.87 (10)	C16—C15—H15	118.9 (11)
C7—C2—C3	108.24 (12)	C14—C15—H15	119.4 (11)
C9—C2—C3	113.77 (10)	C15—C16—C17	118.50 (13)
C4—C3—C2	104.09 (11)	C15—C16—H16	120.4 (9)
C4—C3—H3A	111.4 (10)	C17—C16—H16	121.1 (9)
C2—C3—H3A	107.7 (10)	C16—C17—C12	118.72 (12)
C4—C3—H3B	115.6 (11)	C16—C17—C9	134.95 (11)
C2—C3—H3B	109.1 (11)	C12—C17—C9	106.31 (11)
H3A—C3—H3B	108.5 (14)	N1—C18—C19	116.05 (10)
C5—C4—C3	104.26 (11)	N1—C18—H18A	106.8 (9)
C5—C4—H4A	108.1 (14)	C19—C18—H18A	109.9 (8)
C3—C4—H4A	112.0 (13)	N1—C18—H18B	103.9 (9)
C5—C4—H4B	109.1 (14)	C19—C18—H18B	108.7 (9)
C3—C4—H4B	112.6 (13)	H18A—C18—H18B	111.5 (12)
H4A—C4—H4B	110.6 (19)	C20—C19—C24	118.62 (13)
O6—C5—N1	124.06 (13)	C20—C19—C18	120.58 (11)
O6—C5—C4	126.73 (12)	C24—C19—C18	120.70 (12)
N1—C5—C4	109.21 (11)	C19—C20—C21	120.72 (15)
N8—C7—C2	177.60 (16)	C19—C20—H20	118.9 (10)
C10—C9—C17	107.09 (11)	C21—C20—H20	120.3 (10)
C10—C9—C2	126.59 (12)	C22—C21—C20	120.49 (18)
C17—C9—C2	126.31 (10)	C22—C21—H21	118.2 (14)
N11—C10—C9	109.47 (13)	C20—C21—H21	121.3 (14)
N11—C10—H10	122.1 (10)	C23—C22—C21	119.72 (16)
C9—C10—H10	128.4 (10)	C23—C22—H22	120.8 (12)
C10—N11—C12	109.29 (11)	C21—C22—H22	119.5 (12)
C10—N11—H11	127.3 (11)	C22—C23—C24	120.51 (16)
C12—N11—H11	122.9 (11)	C22—C23—H23	122.7 (14)
N11—C12—C13	129.75 (12)	C24—C23—H23	116.8 (14)
N11—C12—C17	107.83 (12)	C19—C24—C23	119.93 (17)
C13—C12—C17	122.42 (14)	C19—C24—H24	118.7 (10)
C14—C13—C12	117.34 (13)	C23—C24—H24	121.3 (10)

### Hydrogen-bond geometry (Å, °)

**Table:**

*D*—H···*A*	*D*—H	H···*A*	*D*···*A*	*D*—H···*A*
N11—H11···O6^i^	0.90 (2)	2.01 (2)	2.866 (2)	158 (2)

Symmetry codes:  (i) *x*, *y*−1, *z*.

## References

[bb1] Bruker (2000). *SHELXTL-NT* Version 6.10. Bruker AXS Inc., Madison, Wisconsin, USA.

[bb2] De Clercq, E. (1996). *Rev. Med. Virol.* **6**, 97–117. 10.1002/(SICI)1099-1654(199606)6:2<97::AID-RMV168>3.0.CO;2-4 10398452

[bb3] Enraf–Nonius (1988). *CAD-4 Manual.* Version 5.0. Enraf–Nonius, Delft, The Netherlands.

[bb4] Johnson, C. K. (1976). *ORTEPII* Report ORNL-5138. Oak Ridge National Laboratory, Tennessee, USA.

[bb5] Karapetyan, H., Tamazyan, R., Martirosyan, A., Hovhannesyan, V. & Gasparyan, S. (2002). *Acta Cryst.* C**58**, o399–o401. 10.1107/s0108270102009241 12094058

[bb6] Martirosyan, A. O., Gasparyan, S. P., Oganesyan, V. E., Mndzhoyan, Sh. L., Alexanyan, M. L., Nikishchenko, M. N. & Babayan, G. Sh. (2000). *Chem. Heterocycl. Compd.* **36**, 416–419.

[bb7] Martirosyan, A. O., Hovhannesyan, V. E., Gasparyan, S. P., Karapetyan, H. A., Panosyan, G. A. & Martirosyan, V. O. (2004). *Chem. Heterocycl. Compd.* **40**, 1007–1008.

[bb8] Sheldrick, G. M. (1997). *SHELXS97* and *SHELXL97* University of Göttingen, Germany.

[bb9] Spek, A. L. (1997). *HELENA* University of Utrecht, The Netherlands.

[bb10] Tamazyan, R., Karapetyan, H., Martirosyan, A., Hovhannesyan, V. & Gasparyan, S. (2002). *Acta Cryst.* C**58**, o386–o388. 10.1107/s0108270102008636 12094054

